# Supplementary material for: Are carbon emissions trading and green financial instruments synergistic? -Comprehensive quantitative research based on content analysis
Source: PLoS One. 2024 Mar 7;19(3):e0298601. doi: 10.1371/journal.pone.0298601 (PMC10919601; doi:10.1371/journal.pone.0298601)
Supplement: S1 Data — (ZIP) [file pone.0298601.s001.zip › Data/地级市Z_F工作报告.zip!/┴╔─■/▒╛╧¬╩╨/└┤╫╘╣1⁄2╓┌║┼í╛┬φ┐╦╩2╛▌═°í┐_2009.docx]

各位代表：

现在，我代表市人民政府向大会报告工作，请予审议，并请市政协委员和其他列席同志提出意见和建议。

一、2008年工作回顾

刚刚过去的一年，是本届政府的开局之年。这一年，是经济发展面临形势比较严峻的一年，是全市上下付出比较多的一年，更是近年来取得成效比较大的一年。在省委、省政府和中共本溪市委的正确领导下，在市人大和市政协的监督支持下，市政府团结带领全市人民深入学习实践科学发展观，昂扬向上，奋发进取，创业实干，抓项目促发展、抓环境促开放、抓民生促和谐、抓平安促稳定，超额完成了十四届人大一次会议确定的各项任务。预计全年：生产总值实现585亿元，增长15%。规模以上工业增加值完成320亿元，增长18%。地方财政一般预算收入完成42.2亿元，增长25%。全社会固定资产投资实现223亿元，增长35%。社会消费品零售总额实现140亿元，增长21%。城镇居民人均可支配收入实现13300元，增长19.8%。农民人均纯收入实现6170元，增长18%。主要经济指标增幅达到和超过了全省平均水平。

（一）经济结构调整步伐进一步加快

工业强市不断推进。规划实施了总投资391亿元的200个重点技改项目，当年竣工投产100个。当今世界轧幅最宽、轧制水平最高的2300mm带钢热轧机组和东北地区炉容最大的4747立方米高炉在本钢投产运行。北钢1780热连轧生产线建成投产。全市规模以上工业企业达到551户，当年增加100户。钢铁深加工制品业实现增加值17.3亿元，增长25%。非公有制企业增加值完成304亿元，增长25.6%；上缴税金35亿元，增长23.6%。我市正式融入沈抚本国家级高新技术产业带。全年高新技术产业增加值完成56.8亿元，增长28.5%。开发新产品220项，实现销售收入270亿元。新增9个省级名牌产品。特别是在去年第四季度，全力克服宏观经济环境急剧变化带来的不利影响，强化工业经济运行管理与协调服务，本钢和北钢没限量、没减产、没减薪更没有减员，充分体现了国有大企业在关键时候的社会责任和风范。两大钢铁企业全年工业增加值分别实现120亿元和70亿元。工业为全市经济保增长、促发展起到了决定性作用，做出了突出贡献。

药业基地建设开局良好。在八个多月的时间里，编制完成了产业规划、生态规划和起步区控制性详规，6平方公里起步区基本实现“七通一平”。药业基地成功申报为省级生物医药高新技术产业基地。国家综合新药研发大平台获准筹建。46个科技项目得到省科技厅7350万元专项支持。沈阳药科大学、辽宁中医药大学、修正药业、东北制药等签约入驻项目总数70个，投资总额75亿元。药业基地的科技平台和产业集聚初具规模，开发了新的生产力，这必将加速转化为本溪工业的新优势。

现代服务业实现新发展。大商超市转山店和溪湖店、华联商厦嘉业店、铜锣湾银座购物广场等一批大型商场超市建成开业。随着太子城假日酒店的竣工运营，本溪成为辽宁省第4个拥有五星级酒店的城市。全年旅游总收入突破100亿元，增长32.4%；接待国内外游客1959万人次，增长85%。房地产新开工165万平方米，当年竣工170万平方米，在建459万平方米。财税部门加大征收工作力度，地方财政一般预算收入增幅连续四年超过20%以上，收入规模创历史最高水平。全市金融机构各项存款余额和贷款余额分别达到552亿元和382亿元，比去年初分别增加99亿元和100亿元，分别增长21.8%和33.3%。保险业保费收入实现16.7亿元，增长41.5%。现代服务业的发展空间正在不断扩大。

县区域经济发展和新农村建设取得新成果。七个特色工业园区建设进展较快。全面落实各项惠农支农政策，粮食总产27.3万吨，创历史最高水平。新发展中药材、山野菜、干果经济林等43.6万亩。江苏雨润集团在两县投资的肉联加工项目分别开工和签约。北京汇源果品蔬菜加工项目竣工投产。永祥绒山羊二期工程完成厂房建设。26家企业参加第六届北京国际农产品交易会成果丰硕。新完成一批防洪灌溉、人畜饮水、农村能源等基础设施项目。两县四区和开发区生产总值平均增长20%左右，财政一般预算收入平均增长30%以上，县区域经济已成为本溪经济发展的重要支撑。

（二）对外招商和改革效果显著

全年组织开展了12次专题招商活动。共组织15个团组、600多人次，到日本、韩国、新加坡等国家和香港、长三角、珠三角等地区进行了专题概念招商。成功举办了“9?25”香港招商推介会和“9?26”首届生物与医药高新技术交易会，“中国北方药谷、本溪生态新城”在国内外的影响初步形成。筛选确定了投资额千万元以上重点招商项目100个，当年签约落地63个。2008年在国际国内经济形势发生重大变化的情况下，全市实际直接利用外资首次突破1亿美元大关，达到1.13亿美元，增长32.5%；引进域外投资65亿元，增长66%；出口创汇24亿美元，增长94%。不断拓展招商新领域，本溪扩大开放、走出山城迈出了新步伐。同时，国资监管、本钢社会职能移交、集体林产权制度配套改革等各项改革措施不断深化，也为经济社会发展注入了新的生机和活力。

（三）城市发展和环境保护迈上新台阶

城市规划建设水平有新的提升。完成了城市总体规划调整方案，编制了60平方公里的本溪生态新城区发展规划及核心区控制性详规。城市规划展示中心、广播电视数字传输中心、职工培训中心、市博物馆、体育馆、清真寺等标志性公共建筑竣工。本辽辽高速公路通车。沈本产业大道一期建成，二期开工建设。小市至碱厂一级路改扩建工程结束。改造了广裕路、环山路等总长24.1公里的14条市政道路。滨河北路全线畅通。峪溪大桥竣工通车。占地13万平方米的枫叶广场向市民开放。新建和续建了4处太子河滨河公园。北沙河石桥子段建成堤防3.67公里、拦河坝2座。220千伏太子河变电所投入运营。市容市貌又有新的改观。地工路企业搬迁改造完成过半，腾空土地46万平方米。明山粮库迁建完成主体工程。华联商厦的“烂尾楼”修建工程全部完工。城区栽植树木7万株，新增绿化景点30处。楼体亮化和粉饰402栋。在144个住宅区绿化补植乔灌木和草花18万株。对市区24条主次干路进行了综合整治，拆除违章建筑249处。大力开展城乡清洁工程，中心城区道路始终保持清洁率100%。桓仁县争创国家卫生县城通过省级评审。2万平方米以上住宅区物业管理率提高到63%。城市生态环境得到新的改善。正式申报创建国家森林城市。全市森林覆盖率达到73.8%。安排落实了总投资44.4亿元的73个节能减排项目。全市二氧化硫和化学需氧量分别完成减排2.37万吨和1.26万吨。万元生产总值综合能耗下降4.4%。彻底关停了耗能高、污染重的本钢一铁厂。“两钢”和水泥行业共关停了4座高炉、5座焦炉、2台烧结机和8条回转窑生产线。实施了4个污水处理厂和2个城市截流管网建设工程。取缔拆除城区违规锅炉40台、烟囱35根。彻底关闭造纸企业22家、选矿企业47家。全市环境空气质量二级（良）以上天数321天，比上年增加11天。全国113个城市“城考”排名由第72位晋升到第59位。我们可以自豪地说，本溪作为重工业城市，创造了生产快速发展、环境持续改善、人与自然进一步和谐的新业绩。

（四）各项民生工程更多地惠及百姓

全年完成城镇实名制就业7.6万人，城镇登记失业率控制在4.7%。“零就业家庭”持续保持动态为零。安置棚户区回迁下岗失业人员3966人。实名制劳务输出31923人。新开发公益性岗位3000个。“五项保险”新增扩面13.9万人。17.6万名企业离退休人员养老金全部按时足额发放，发放标准月人均提高100元。城镇居民基本医疗保险参保人数达到17万人，参保率50%。新农合筹资标准提高到每人每年100元，报销封顶线提高到3万元。城镇失业保险金月人均发放标准提高到280元和320元。城市职工月最低工资标准提高到580元。11.2万人城乡低保做到了持续应保尽保，月人均救助额分别提高到160元和68.2元。建立完善了城乡低保边缘户救助制度。“两节”期间共筹集资金1900多万元，救助城乡困难群众7.5万人次。农村五保对象供养标准提高到2900元。

基本完成5万平方米以下棚户区改造，安置居民9730户。安排经济适用住房14万平方米，新建廉租住房2.1万平方米，为865户困难群众发放租赁补贴152.9万元。为农村特困户建房735户。维修了150条街巷路。亮化了115条“摸黑路”。修建了103个住宅区内的人行甬道和车行道16.9万平方米。提前7个月取消滨河南路收费。改造了本溪火车站。城市供暖质量稳步提高。全市基本实现了村村通油路。新建成30处村村通自来水工程。南芬区10万人口多年企盼的生活饮用水问题得到彻底解决。

落实义务教育“两免三补”、高中阶段“宏志生”和中职学生助学专项资金6244万元，免除行政事业性收费122万元，受益学生14.6万人。解决了中小学教师工资待遇保障问题。改造建设了10处集贸市场。大河市场农产品检验站投入使用。加强市场价格监管和调控，居民消费价格指数增幅低于全省平均水平。

经过一年的努力工作，年初向全市人民郑重承诺的十项民生工程全部兑现落实，全市各项社会保障标准都已经提高到全省平均水平以上。我们就是要这样一年一年地做下去，让山城百姓不断切身体会到，我们的政府是倾心为民的政府。

（五）社会稳定和谐的局面更加巩固

强化了安全生产监管和隐患排查，全市共查出各类事故隐患9127项，整改结束9053项，限期整改74项。彻底关闭了两家烟花爆竹生产企业。专项整治了煤矿、非煤矿山尾矿库、危险化学品以及道路交通和消防安全隐患，全市安全生产工作在省政府目标管理考核中位居前列。深入开展信访突出问题“三大战役”和重信重访专项治理活动，稳妥地处置了“蚁力神”等涉众事件，一大批涉及民生的重点信访问题得到妥善解决。全年受理信访总量40911件（次），下降16%。排查调解各类矛盾纠纷6126件，调解成功率97%。圆满完成了奥运安保任务，市公安局荣获“全省奥运安保先进集体”光荣称号。全面加强社会治安巡逻防控体系建设，深入开展了“打黑除恶”以及“抓逃犯、破积案、打盗抢”等系列专项行动，全市刑事、治安案件大幅度下降，广大群众的安全感进一步增强。特别是“5?12”四川等地发生强烈地震后，全市各族人民积极行动，奉献爱心，累计捐赠款物6940.9万元。我市先后向灾区派出抗震救援小分队、特警工作队、医疗队和卫生监督防疫队7批次、128人。市级行政机关和事业单位还节省5%的预算经费支出，用于支援灾区重建。事实证明，本溪人民顾全大局，本溪人民富有爱心，本溪人民乐于奉献，这是推进本溪振兴发展与和谐社会建设的强大力量。

（六）各项社会事业进一步协调发展

优先发展了教育事业。加大对中小学基础设施建设的投入，推进了义务教育区域内均衡发展。整合职业教育资源，组建了市机电工程学校。本钢工学院与辽宁冶金技师学院合并申办高等职业技术学院。高考总录取率达到84%，41人考入清华北大。发展繁荣了文化事业。现代评剧《女人是座山》获国家级大奖。成功举办了庆祝本溪解放六十周年和改革开放三十周年系列文化活动。“本溪鼓乐”等3个项目被列入国家级非物质文化遗产保护名录。着力提高了医疗公共卫生服务水平。出色地完成了奥运期间医疗卫生保障、支援地震灾区医疗救援任务。妥善处置了手足口病疫情、食用含三聚氰胺奶粉等突发公共卫生事件。市传染病医院和结核病院成功合并，传染病医院二期工程开工建设。建成6所社区卫生服务中心和53所村卫生室。组织开展了“百名医疗专家下基层”活动。全面推进了体育等各项事业健康发展。奥运火炬传递筹备工作得到国家和省的表扬。与奥运同行的群众性体育活动丰富多彩，竞技体育获得省以上金牌109枚。广播电视完成了主城区范围的数字化整体转换，桓仁县城转换任务也基本结束。持续稳定了低生育水平，人口自然增长率控制在0.99‰以内，兑现了计生奖扶政策。第二次经济普查全面铺开。顺利完成197个城市社区的集中换届，在95个村实施了农村社区建设试点。民族宗教、人防、外事、侨务、档案、地方志和气象、防震、优抚及社会福利、老龄、慈善、残疾人事业等也取得了新成绩。

在工作中，市政府始终坚持把加强自身建设放在重要位置，全面落实依法行政责任制，不断推进了服务型政府和法治政府建设。提请市人大制定地方性法规2件，制定政府规章5件。编制印发了《市政府信息公开指南和目录》。第五轮行政审批制度改革清理审批事项405项。全年办理人大代表建议和政协提案993件，办复率100%、解决率67.1%。开通了县区长和委办局长（主任）信箱。市长公开电话和市长信箱保持昼夜畅通，分别受理市民投诉12008件（次）、网上信件1736件。强化行政监察和审计监督，提高了廉洁从政水平和财政资金的绩效。市直59个单位共申报服务成果121件，53件被评为最佳服务成果。市公安局开展的“千名民警进万家”活动经验在全省和全国推广，本溪广大公安干警用实际行动进一步密切了警民关系，更密切了党和政府与人民群众的血肉联系。

各位代表，刚刚过去的一年，我们立足振兴，在宏观环境复杂多变的情况下，坚定信心，昂扬向上，奋发作为，保持了本溪经济社会发展的良好势头；我们突出发展，以项目年为载体，围绕基础设施、工业、农业和服务业，规划推进了一大批项目；我们关注民生，从群众最关心、最直接、最现实的利益问题出发，妥善解决了一大批改革发展中的问题；我们着眼长远，努力改善发展环境，保护生态资源，为实现本溪可持续发展奠定了坚实基础。实践已经并将继续证明，无论在什么形势下，我们本溪的干部群众是能够打硬仗的，是有战斗力的，是能够经得住任何困难考验的，本溪未来的发展更是大有潜力和希望的。

过去一年的发展和进步，是省委、省政府和市委正确领导的结果，是市人大、市政协监督支持、团结协作的结晶，凝聚了全市各族人民的智慧、心血和汗水。在这里，我代表市政府，向辛勤工作在各行各业的全市人民，向给予政府工作大力支持的各级人大代表和政协委员、各民主党派、工商联、无党派人士、各人民团体和社会各界人士，向驻溪解放军、武警部队官兵表示衷心的感谢!

在肯定成绩的同时，我们也清醒地认识到，目前全市经济社会发展确实还存在着一些突出矛盾和问题：一是我市以钢铁为支柱产业，结构比较单一，接续产业还没有形成接续能力，经济增长方式仍然粗放，资源利用水平偏低。二是影响社会和谐稳定的因素仍然存在，部分群众在生活、就业、就医、社保、住房等方面还存在许多实际困难，城市供暖、供气、给排水等设施历史欠账较多。三是农业和农村基础设施建设还比较薄弱，农民增收难问题还比较突出。四是政府部门的工作效率不够高，思想不够解放，开放招商的软环境建设还有不少差距。特别应当指出的是，当前由美国次贷危机引发的金融危机的影响，已经由虚拟经济转向实体经济。从本溪产业结构分析，钢铁产品和装备制造业部分产品的需求可能持续减少，价格还要下滑，出口会明显萎缩，两大钢铁企业和与之配套的中小企业生产经营面临的困难会更多。但更应当看到，本溪经济社会发展的主要层面并没有变，总体上仍然是机遇与挑战并存、机遇大于挑战。当前遇到的这些困难和问题，都是前进中的困难、发展中的问题，我们有信心、有能力去不断克服和解决。

二、2009年主要工作任务

今年是实施“十一五”规划的关键一年。尽管受金融危机冲击影响的因素还不能最后确定，但只要我们有充分的思想准备和应对措施，全市上下坚定信心，迎难而上，善于把危机变为契机，善于把危机化为转机，扩内需、抓投资、上项目、调结构、保增长、积后劲、促和谐，就一定能够把本溪现在的事情办好，把本溪的未来建设得更好。

今年全市经济社会发展的总体要求是：全面贯彻落实党的十七大和十七届三中全会精神，以邓小平理论和“三个代表”重要思想为指导，深入学习实践科学发展观，以经济保增长、民生保就业、社会保和谐为目标，继续大力实施三大结构调整、工业强市、外向牵动和人才兴市战略，更加注重调整经济结构和转变发展方式，更加注重项目建设和开放招商，更加注重资源节约和环境保护，更加注重改善民生和促进社会和谐，确保全市经济社会持续平稳较快发展。

全市经济发展的主要预期目标是：生产总值和地方财政一般预算收入均要增长13%，固定资产投资增长30%，城镇居民人均可支配收入和农民人均纯收入均增长12%。在实际工作中，我们还要力争主要经济指标增幅达到或超过实施振兴战略以来的平均水平，达到或超过全省的平均水平。

坚定不移、毫不动摇地实现上述目标，打好今年的攻坚战，我们必须清醒认识和充分利用好各种积极因素与有利条件：中央着力扩大内需、实施积极的财政政策和适度宽松的货币政策，为我们创造了良好的宏观环境；全省加快推进沈阳经济区建设、举全省之力支持本溪生物医药产业发展，为我们带来了千载难逢的发展机遇；特别是在中共本溪市委的正确领导下，全市上下政通人和，发展思路更加清晰，发展目标更加明确，发展信心更加坚定，谋发展、促振兴的氛围更加浓厚。我们一定能够顶住压力、克服困难，在现有的基础上实现更快的发展、更大的进步。

新的一年要重点做好以下工作：

（一）落实扩大内需方针，抓好基础设施投资项目。今明两年是本溪市历史上基础设施投入最大的时期。对规划确定的重点项目，要明确负责领导和承办单位，落实目标责任和工作措施，全力以赴地跟踪推进。要积极争取桓仁核电项目列入国家核电发展规划。本溪热电厂要获得国家核准并开工建设。两县热电厂要完成前期准备工作并报国家核准。桓仁抽水蓄能电站和三个风电厂争取开工建设。500千伏程家输变电工程年底竣工。开工新建和扩建总投资19亿元的36个变电工程（所）项目。加快推进沈本城际轨道交通前期工作，确保准时开工建设。东北东部铁路桓仁段要加快建设进度。田师付经桓仁到灌水铁路要具备开工建设条件。争取将溪田线城市段改线列入铁道部规划。鹤大高速和桓永高速公路桓仁段要加快建设进度。沈本高速公路高台子出口要开工建设。沈本产业大道二期实现竣工通车。推进桓仁支线机场前期工作。此外，还要切实策划、论证、包装好“十二五”项目。这些打基础、增后劲、促长远的事情，我们要持之以恒、坚持不懈地干下去。

（二）保持工业经济稳定增长，加大药业基地建设力度。越是在困难的时候，我们越要坚持发展是硬道理的战略思想。工业是本溪保增长的主导产业。要全力支持“两钢”搞好生产经营和重大项目建设。本钢要加快超薄冷轧板二期、三冷轧、新2号高炉、不锈钢冶炼及冷轧等项目建设步伐，确保2011年建成投产。北钢要开工建设300平方米烧结机改造工程，推进干熄焦装置及老焦化改造。继续加大钢铁深加工制品产业发展力度，拉长钢铁产业链。抓紧推进多晶硅太阳能电池项目和4条新型干法水泥熟料生产线项目。继续滚动实施好200个技改项目，确保当年竣工投产100个。抓住机遇加快结构调整，支持重点技改项目和节能改造项目建设，大力开发高附加值、高科技含量的产品，推进实施名牌战略。加强煤、电、油、运等生产要素的协调供应，强化企业管理，降低产品成本，不断开拓新市场，保持工业经济稳定运行。全市规模以上工业增加值要增长18%以上。继续开发200项新产品，新产品销售收入要实现300亿元。培育和认定25家市级以上高新技术企业和企业技术研发中心。全年高新技术产业增加值要增长25%。推进银企对接，促进中小企业、促进非公有制经济保持快速发展势头。全市非公有制企业增加值和上缴税金均要增长20%。要因势利导、乘势而上，进一步加快打造“中国北方药谷”。强化药业专项招商，全年新增落地项目100个，新增投资额100亿元。积极申报国家级生物医药高新技术产业基地。继续加大药业基地基础设施建设力度，营造药业招商软环境，加快入驻项目开工建设和投产达效，让落地的种子真正开花结果，今年“北方药谷”要有产出、要有产品、要有成果。我们最终期待的是本溪药业发展硕果累累的秋天。

（三）全面推进改革开放，为加快发展增添新活力。要落实好有利于实现保增长、扩内需、调结构的改革措施，不断深化水、气、热等价格体制改革。建立国有资本经营预算制度，全面推行国有企业经营业绩考核，促进38户国有独资和控股、参股企业做大做强。完成地工路企业整体搬迁任务。基本完成24户企业破产终结。着力推进聚丰公司上市，做好冶炼集团等企业上市准备。积极稳妥地推进集体企业改革。围绕深化行政管理体制改革的总体目标，扎实推进政府机构改革。要主动承接国际国内特别是长三角、珠三角的产业资本转移，采取重点招商、网上招商、驻在招商等有效方式，吸引南资北上、南企北上，吸引符合我市发展的产业项目入驻。在抓好百日招商活动的基础上，继续保持对外招商的频率和次数。举办好第二届辽宁?本溪生物与医药高新技术交易会。策划包装好新100个投资额千万元以上的重点招商项目。积极引导企业调整产品结构、开拓新的国际市场，支持“两钢”出口。全年实际直接利用外资和引进域外资金均要增长20%，出口创汇额要保持在14亿美元以上。要通过扩大开放招商，增加发展的机会和资本，增加发展的项目和人才，使本溪的项目更多、企业更多、经济增长点更多。

（四）着力解决制约发展的要素问题，科学配置人才、土地和资金。采取市场配置、项目集聚、外埠招聘等多种方式，重点引进60名左右支柱产业和接续产业发展急需的高层次人才。推进海外研发团队项目实施工作，组织实施专家项目40个、聘请外国专家60人次以上。科学合理地修编土地利用规划，以新区、药谷和各县区工业园区建设为战略重点，着力抓好药业基地以及姚家、威宁一带新区用地的调整、申报和征地拆迁。研究推进不受行政区域限制，扩大药业基地发展空间。努力盘活存量土地资源，大力推广节约集约用地。积极向上争取国债和专项资金，千方百计拓宽融资渠道。加强与国家开发银行、农发行和各商业金融机构的合作，支持鼓励金融机构加大信贷投入，保证重点项目建设、企业生产经营的资金需求。积极争取引进一家股份制银行。城信社转制为商业银行。大力推进小额贷款公司和村镇银行试点，每个县区都要建立1个中小企业信用担保中心和1、2个小额贷款公司。研究实施发行地方政府债券和企业集合债券。切实加强税收和非税收入征收管理，强化财政经费定额和项目预算管理，扩大政府采购范围。要科学整合财力资源，进一步调整支出结构，保重点、保民生、保稳定。要大力压缩一般性支出，始终坚持勤俭办事业，确保财政收支平衡，全市上下都要有压缩支出、过紧日子的思想和实际行动。

（五）不断壮大县区域经济，扎实推进新农村建设。坚持统筹城乡发展，加大以工补农和以城带乡工作力度，全面推进农村工业化、城镇化和产业化。七个工业园区要注重发挥比较优势，加快基础设施建设，扩大开放招商，尽快形成各具特色的产业集群。今年要下力气抓好一大批农产品深加工企业。着力推进沿本桓公路两侧的50个现代农业和产业化重点项目。扶持10家农业科技星火龙头企业。突出发展精品农业、高效农业和设施农业。新建林下参、五味子、刺五加等中药材基地9万亩。建成150个设施农业小区、150个畜禽标准化养殖小区。建立健全农业科技推广和服务体系，发展农民专业合作社，推进农村土地流转市场建设。继续深化集体林产权制度配套改革。扩大政策性农业保险覆盖面，化解农业灾害损失。开工建设观音阁引水工程。继续实施太子河、北沙河、细河等河道治理。新建农田灌溉和农村安全饮水工程140处（项），其中村村通自来水22处。县区生产总值和财政收入平均增幅均要达到20%以上，促进壮大全市经济规模和实力。

（六）加快发展现代服务业，为保增长做出更大的贡献。大力推进和开工建设沃尔玛超市和新玛特商场等19个商贸流通重点项目。策划包装一批房地产开发项目，积极引进国内外大型开发商进入本溪市场，重点推进地工路、原广电局、明山粮库等地块的开发建设。全市全年新开工300万平方米、竣工240万平方米。两县要加大县城房地产市场开发力度，新开工和竣工面积都要达到30万平方米。去年出台的支持房地产业发展的优惠政策延长到今年年底。要为旅游业形成支柱产业不断创造条件。重点推进温泉小镇规划建设、桓仁八卦城恢复、五女山高句丽景观园和关门山、铁刹山风景区扩大改造等项目。积极推进本溪水洞申报国家5A级景区和世界自然遗产。研究整合旅游资源，逐步理顺管理体制。抓住“2009中国生态旅游年”契机，强化宣传促销，深化区域合作，打造知名品牌，完善精品旅游线路。全年接待游客和旅游总收入要分别增长35%和30%。全市服务业增加值和社会消费品零售总额要分别增长20%和15%，努力使服务业成为保增长、促发展的重要力量。

（七）切实抓好节能减排，进一步改善生态环境。认真做好能源评估、能源审计和能源监查工作。加快推进33个重点节能和资源综合利用项目建设。万元生产总值综合能耗要下降4.4%。继续争创“全国环保模范城”，深入开展“环保攻坚惠民”活动。实施好28个重点减排和污染源治理项目。搬迁改造本溪水泥厂。二氧化硫和化学需氧量要分别完成减排1.54万吨和0.47万吨。继续加强太子河、细河流域的污染治理和环境监管。南芬区、开发区和北钢污水处理厂要建成运行。市污水处理厂扩容升级工程要开工建设。建设千金沟等3个城市排污沟截流工程。建成千金岭垃圾处理场。全年城市环境空气质量二级（良）以上天数要达到320天以上。严格执行退耕还林政策，坚决制止“小开荒”。严厉打击乱采矿产资源、破坏森林资源的违法行为，保持住73.8%的森林覆盖率。通过扎实工作，持续为山城人民保留好一方青山绿水、撑起一片蓝天。

（八）强化城市规划建设和管理，不断提升城市品位。投资3.5亿元实施平山横三路、柳塘路等7条市政道路改造。开工建设北地跨线桥。建成大峪新区育龙路。对体育路等3条道路实施综合整治。新栽各种树木7万株，新增20栋楼体亮化工程。改造老城区超期使用的供水和供暖管网。加快创建国家森林城市，在城周山有计划地进行生态风景林建设，全年人工造林10万亩、新增封山育林10万亩，尽快形成“外部森林环抱、内部绿树成荫”的城市森林格局。继续深入开展城乡清洁工程，整治市容环境，强化日常管理。出台《本溪市物业管理条例》，积极推进多种形式的物业管理。支持客运集团更新公交车辆。加快推进本溪新城区的开发建设，在药业基地起步区形成畅通的道路网络，完善供水、排水、供暖、电力以及污水处理等基础设施。我们要通过卓有成效的工作和坚持不懈的努力，使本溪的城市规划建设和管理水平再上一个台阶，城市结构布局的调整加快实施步伐，硬环境更加适宜经济发展和市民居住，让群众对本溪城市的未来更加充满信心。

（九）继续改善民生，提高群众收入水平和生活质量。改善民生是保增长的出发点和落脚点。今年民生工作的重点主要是增加就业。要不停顿地组织各种形式的就业洽谈会，提高稳定就业比重。城镇实名制就业要达到6.8万人，登记失业率要控制在5%以内，还要动态储备一批就业岗位。扶持创业带头人340人，带动就业2040人。实名制劳务输出2万人。继续保持“零就业家庭”的动态为零。企业退休职工养老金月人均再增加100元。城镇居民基本医疗保险参保面要达到80%以上。全部解决集体困难企业退休人员基本医疗保险问题。积极推进农民工参加养老保险。继续推进企业职工工资集体协商制度。适时提高企业工伤人员伤残津贴、生活护理费和供养亲属抚恤金标准。城乡低保要继续实现应保尽保。进一步提高优抚对象抚恤和生活补助标准。新农合报销比例在现有基础上再提高10%，封顶线要由3万元提高到4万元。继续加大保障性住房建设力度。为农村贫困户建房260户。新建和改造4所中心敬老院。将低保户和低保边缘户住房租赁补贴标准分别提高到240元和120元。再改造一批老城区的街巷路和“摸黑路”。基本结束主城区住宅小区人行甬道和车行道的改造。开通本溪主城区到石桥子的公交车。在实际工作中，我们还要尽最大的能力，为老百姓办更多的实事，让群众得到更多的实惠。

（十）大力发展各项社会事业，努力营造更加和谐稳定的社会环境。推进义务教育均衡发展，完成本溪县、南芬区和开发区“双高”“普九”达标任务。开工建设本溪市高级中学石桥子分校。完成机电工程学校新校舍主体工程和化校实训基地建设。启动沈阳药科大学职业技术学院搬迁。支持辽宁科技学院新校区建设和老校区开发。继续落实好“两免三补”和“宏志生”、中职生资助等政策。推进市档案馆以及钢铁、药业和水利工程三大博物馆规划建设。办好本溪市第十二届文化艺术节。继续抓好农村公共文化服务“五项工程”建设。建设市中医院综合门诊楼。新改扩建10所社区卫生服务中心和80所村卫生室。传染病医院二期工程竣工并投入使用。继续深入开展“百名医疗专家下基层”活动。办好市第九届全民运动会。抓好市体育馆设施完善和运行管理。全面完成本溪县城和南芬主城区广播电视数字化整体转换。推进文化信息资源共享工程进村入户。稳定低生育水平，统筹解决人口问题，人口自然增长率控制在1.31‰以内。全力做好第二次经济普查。加强国防后备力量和人民防空建设，继续做好双拥共建工作。争创全国民族团结进步模范市“五连冠”。两县要办好满族自治县成立20周年庆祝活动。大力发展慈善、红十字和残疾人等事业。深入开展“五五”普法依法治理活动。全面提高城市社区为民服务水平。建成90个农村社区。毫不放松地抓好安全生产。进一步加强食品药品监督管理。搞好粮食储备，确保粮食安全。健全社会预警体系，切实提高公共安全保障和处置突发事件的应急能力。深入开展领导干部下访活动，建立健全行政调解、司法调解和人民调解相衔接的协调运行机制，妥善处理和积极化解各类矛盾纠纷。全面落实社会治安综合治理的各项措施。深化开展“千名民警进万家”活动，继续大力推进“平安本溪”建设，进一步完善社会治安巡逻防控网络体系，严厉打击各种违法犯罪活动，为人民群众营造更加安居乐业的社会环境。

各位代表，面对国际国内形势的新变化，面对众多不可确定的新因素，完成今年的目标任务光荣而艰巨。困难就是考验，挑战就是锻炼。每个政府公务员特别是各级领导干部都要进一步解放思想，把思想真正统一到科学发展观的要求上来，统一到“保增长、扩内需、调结构”的目标上来，统一到真抓实干、昂扬奋进的精神状态上来，立足本职，主动想事，秉公办事，大胆管事，扎实做事，全力做到谋发展的势头不减、促发展的干劲不减、保发展的指标不减。要通过深入学习实践科学发展观的活动，在心态上、能力上和作风上实现新的适应和新的提升。一是再勤奋学习。要大力提倡勤奋学习的风气，每个人都要通过认真学习政治理论、现代经济、科技、社会管理和法律知识，不断提高驾驭市场经济、依法管理社会的能力，增强为人民群众服务的本领。二是再深入实际。要扎实开展“党员干部走进千家万户”和“解难题、促和谐”活动，真正走近群众、服务群众，解决实际困难。对群众合理要求的，最小的事也要精心去做；对群众反映强烈的，最难的事也要全力去办；对群众不满意的，最棘手的事也要坚决纠正。三是再集思广益。要更加主动地接受人大的依法监督和政协的民主监督，主动接受社会公众和新闻舆论的监督。认真办理人大代表建议和政协提案。加强同民主党派、工商联、无党派人士和人民团体的联系。发挥好市长公开电话和市长信箱的作用，进一步畅通民意渠道，继续贯彻市县政府依法行政决定，推进决策的科学化、民主化和制度化。四是再求真务实。要更加注重求实务实。在工作中，要不讲困难只讲机遇、不讲客观只讲主观、不讲严峻只讲作为，要敢于承担责任、敢于承担风险、敢于触及矛盾、敢于迎难而上，始终保持昂扬向上的精神状态，始终保持埋头苦干的工作劲头，始终保持求真务实的良好作风，真正把主要工作抓到细枝末节、抓到底、抓出成效。要强化政务督察、行政监察和审计监督，不断推进政府的廉洁高效。五是再注重长远。在做好当前工作的同时，更要谋划好未来发展。多干打基础的事，多干若干年后能够受益的事。为了长远利益和全局利益，宁可牺牲眼前的利益和局部的利益，这样才能使本溪经济社会发展的速度更快、活力更强、后劲更足。

各位代表，让我们在中共本溪市委的正确领导下，团结和带领全市各族人民，把握新机遇，迎接新挑战，谱写新篇章，为实现全市经济社会持续平稳较快发展而努力奋斗。
